# Analysis and knowledge extraction of newborn resuscitation activities from annotation files

**DOI:** 10.1186/s12911-024-02736-4

**Published:** 2024-11-05

**Authors:** Mohanad Abukmeil, Øyvind Meinich-Bache, Trygve Eftestøl, Siren Rettedal, Helge Myklebust, Thomas Bailey Tysland, Hege Ersdal, Estomih Mduma, Kjersti Engan

**Affiliations:** 1 Department of Electrical Engineering and Computer Science, University of Stavanger, Stavanger, Norway; 2 Laerdal Medical, Stavanger, Norway; 3 Stavanger University Hospital, Stavanger, Norway; 4 Haydom Lutheran Hospital, Haydom, Manyara Tanzania; 5 Department of Medical Informatics, University of Amsterdam, Amsterdam, The Netherlands

**Keywords:** Newborn, Resuscitation activities, Visualization, Dimensionality reduction, Autoencoder

## Abstract

Deprivation of oxygen in an infant during and after birth leads to birth asphyxia, which is considered one of the leading causes of death in the neonatal period. Adequate resuscitation activities are performed immediately after birth to save the majority of newborns. The primary resuscitation activities include ventilation, stimulation, drying, suction, and chest compression. While resuscitation guidelines exist, little research has been conducted on measured resuscitation episodes. Objective data collected for measuring and registration of the executed resuscitation activities can be used to generate temporal timelines. This paper is primarily aimed to introduce methods for analyzing newborn resuscitation activity timelines, through visualization, aggregation, redundancy and dimensionality reduction. We are using two datasets: 1) from Stavanger University Hospital with 108 resuscitation episodes, and 2) from Haydom Lutheran Hospital with 76 episodes. The resuscitation activity timelines were manually annotated, but in future work we will use the proposed method on automatically generated timelines from video and sensor data. We propose an encoding generator with unique codes for combination of activities. A visualization of aggregated episodes is proposed using sparse nearest neighbor graph, shown to be useful to compare datasets and give insights. Finally, we propose a method consisting of an autoencoder trained for reducing redundancy in encoded resuscitation timeline descriptions, followed by a neighborhood component analysis for dimensionality reduction. Visualization of the resulting features shows very good class separability and potential for clustering the resuscitation files according to the outcome of the newborns as dead, admitted to NICU or normal. This shows great potential for extracting important resuscitation patterns when tested on larger datasets.

## Background

Deprivation of oxygen during and after birth leads to birth asphyxia, a condition recognized as a prominent factor in newborn mortality, as well as in the occurrence of cerebral palsy, learning disabilities, and other developmental delays [1, 2]. To minimize the risk and effect of birth asphyxia, it is important to resuscitate a non-breathing newborn as quickly and efficient as possible. Approximately 85% of term newborns start breathing within 10-30 sec without help, 10 % will respond to stimulation and drying, but around 5-6 % needs ventilation (including positive airway pressure (CPAP) and positive pressure ventilation (PPV)) [3, 4].

Guidelines for newborn resuscitation have been established by the world health organization (WHO) and the International Liaison Committee on Resuscitation (ILCOR) [3, 5]. The guideline advices to start the newborn resuscitation with ventilation within the first minute (golden minute) for a non-breathing newborn [6]. The guidelines are mostly based on best practice and more evidence based research on newborn resuscitation is highly sought for [7]. The main therapeutic measures during newborn resuscitation include ventilation, stimulation, and suction [8, 9]. Other activities that occur less frequent and/or has less direct therapeutic effect include chest compression, drying, warming, epinefrine injections and others [2, 10].

In practice, disparities or variations might arise between guidelines and the actual conducted procedures. Factors such as stress, inadequate training, equipment and staff shortages, physical distances, non-optimal procedures, like delayed resuscitation initiation or insufficient resuscitation duration, can contribute to these discrepancies [11]. To evaluate whether the resuscitation complies with the guidelines and/or if revision of guidelines should be considered, it is necessary with a comprehensive analysis of newborn resuscitation activities coupled with the status of the newborn after resuscitation.

NewbornTime^1^ is a collaborative project, titled “Improved newborn care based on video and artificial intelligence” [12], and the primary objective is to automatically generate an objective timeline describing the activities during newborn resuscitation, including the time of birth (ToB). The data measured for producing the timelines include ordinary (visual light) video over resuscitation tables and thermal video at the labour room. NewbornTimes objectives include the utilization of artificial intelligence (AI), in terms of machine learning (ML) techniques, to objectively detect the resuscitation activities and ToB, producing the resuscitation timeline automatically [12–14]. In clinical practice ToB is usually manually recorded with minute precision. By identifying the ToB automatically with second precision, it becomes possible to ascertain whether resuscitation was initiated during the critical “golden minute” or if there was a delay in the overall resuscitation process.

The goal of NewbornTime is to produce the timeline automatically, but as a step on the way resuscitation timelines can be produced by clinicians manually observing and delineating the recorded videos retrospectively [2]. This gives high-quality labelled data to use when learning AI systems, but it also gives timeline descriptions of the newborn resuscitation that we can start to analyze. The data used in this work originate from video recordings of the resuscitation table. The manual annotations studied in this paper are made using the ELAN video-annotation tool, manually watching the video-recordings, producing text files. In future work, when we have fully functional automated activity recognition models, we will analyze a larger set of episodes with automated timelines. In this paper we focus on the method for extracting knowledge from the available manual annotated file.

**Fig. 1:**
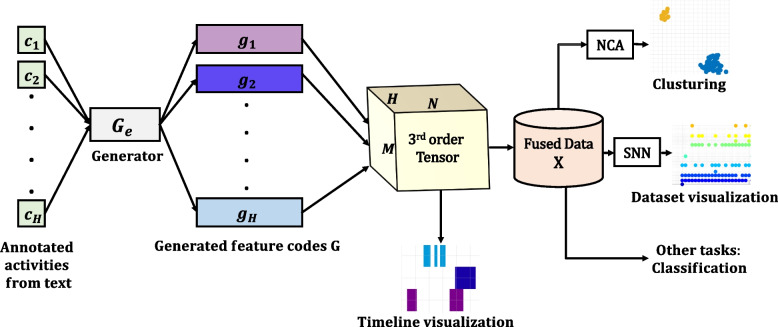
The proposed approach for information extraction and the execution of machine learning tasks, encompassing dimensionality reduction and visualization processes

Knowledge extraction is the process of automatically gathering structured information from possible unstructured sources of data. Extracted knowledge can for example be used for data analysis and data mining tasks [15–17]. In this paper, we propose to utilize knowledge extraction and data mining methods for encoding and visualizing the temporal data extracted from the newborn resuscitation annotation text files, and to discover pattern trends and get insights from a larger batch of resuscitation episodes. An overview of the approach presented in the paper can be seen in Fig. 1.

Somewhat related research can be seen in Sheikhtaheri et al. [18] predicting outcome of newborns admitted to the Newborn Intensive Care Unit (NICU) using machine learning. However, they are using clinical variables and not a description of resuscitation activities as functions of time. In Urdal et al. [19] signal processing and machine learning are used to find important parameters for survival after newborn resuscitation. Here, it is shown that in addition to clinical parameters such as birth weight and gestational age, therapeutic strategies, i.e., resuscitation activities, are important for newborn survival. However, specific features are used, not the entire description of the combination of activities. We want to explore whether we can encode the entire timeline information in a way that is useful to identify good patterns of activities.

There are two main contributions: *(i)* the introduction of a method that encodes the annotation of resuscitation episodes by using a periodic encoding generator, facilitating further data mining. *(ii)* Redundancy reduction is proposed through a learned autoencoder (AE), followed by a neighborhood component analysis (NCA) for dimensionality reduction. The AE-NCA is visually shown to have much better class separability than NCA alone. In addition, a straightforward timeline visualization with and without ToB is presented, and visualization of batches of episodes.

## Methods

In this section, we present the proposed encoding methodology, redundancy reduction and visualization derived from textual data pertaining to resuscitation activities, which correspond to the annotations of resuscitation videos. We begin by formalizing the core concepts underlying our approach in the Notations and formalization section. Subsequently, we provide a comprehensive description of the dataset employed in our study in the Dataset section. We introduce the proposed encoding generator method in the Proposed periodic encoding methodology section. Furthermore, we introduce the redundancy and dimensionality reduction through the incorporation of NCA and AE techniques, which contribute to the visualization of the dataset. The framework is illustrated in Fig. 1.

### Notations and formalization

Because knowledge extraction, representation learning, dimensionality reduction, and visualization have been explored and applied in various fields, the terminologies and mathematical notations can vary, even when referring to the same methods and concepts. Therefore, we define the notation used in this paper in this subsection, and it is listed in Table 1.

**Table 1:** List of notations and mathematical symbols used in this paper

Notation	Description	Notation	Description
*C*	A list of resuscitation activities	$G_e$	Periodic encoding generator
*G*	A matrix of generated feature codes	$g_i$	Generated feature vector\ code
$X \in \mathbb {R}^{N\times M}$	Fused data matrix	$Z \in \mathbb {R}^{d\times M}$	A compact\ latent\ embedded version of *X*
*N, d*	Dimensionality of the data and latent spaces	*H*	The number of main resuscitation activities
*N*	The length of the activ generated feature code	*M*	The number of dataset episodes
$f: \mathbb {R}^N \rightarrow \mathbb {R}^d$	Mapping or encoding function	$\tilde{X}$	Reconstructed data after learning
$g: \mathbb {R}^d \rightarrow \mathbb {R}^N$	Reconstruction or decoding function	$\mathcal {L}$	Optimization target
$\mathcal {G} \in \mathbb {R}^{H \times N \times M}$	$3^{\text {rd}}$-order tensor	*Q*	Orthogonal learnable metric distance
$P_{ij}$	Probabilistic softmax distance	*Y*	Class labels
$\theta _e$	A set of encoding weights, $W_e$, and biases $B_e$	$\theta _d$	A set of decoding weights, $W_d$, and biases $B_d$
*A*	Transformation matrix	$\eta$	Learning rate

In this paper, we analyze annotation text files, where each file corresponds to a single resuscitation episode, $E_{j}$. Let *C* be a set of *H* types of (the main) resuscitation activities $c_{i}$:

1 $$\begin{aligned} C = \{c_i \, , i = 1\, ,\dots ,\, H\} \end{aligned}$$

Within one specific resuscitation episode, $E_{j}$, of length *N* seconds, each activity, $c_i$, can occur multiple times with different duration, and multiple activities can occur at overlapping time-intervals.

For $E_{j}$ we generate a set of feature code vectors, $g_{i}$, of length N, indicating at second precision when activity $c_{i}$ takes place. For each episode $E_{j}, j= 1 \ldots M$, we define a matrix $G_{j} \in \mathbb {R}^{HxN}$ with $g_{i}$ as the $i^{th}$ row:

2 $$\begin{aligned} G_{j}=\left[ g_{1} \ldots g_{H}\right] ^{T} \end{aligned}$$

If $G_{j}(n,i)\ne 0$ it means that for episode *j*, at timepoint *n*, activity $c_{i}$ is being performed. Finally, the $G_{j}$ for all *M* episodes are stacked to form a $3^{\text {rd}}$-order tensor $\mathcal {G}\in \mathbb {R}^{HxNxM}$.

To perform various machine learning tasks such as visualization, dimensionality reduction, and AE learning (as depicted in Fig. 1), and to illustrate the occurrence of activities across all dataset episodes, we introduce the concept of fusion through addition among the generated feature codes of each sub-matrix, $G_j$. This fusion process results in a 2-D fused data matrix denoted as $X \in \mathbb {R}^{NxM}$, where *M* is the number of resuscitation episodes in the dataset. The fused vectors $x_{j} \in \mathbb {R}^{N}$ in the columns of *X*, is one for each episode.

3 $$\begin{aligned} X=[x_{1} \ldots x_{M}], \;\; \text {where} \;\; x_{j}^{T}=\textbf{1}^{T}_{H}G_{j} \end{aligned}$$

$\textbf{1}_{H}$ is a size *H* column vector of ones.

We employ an AE to reduce theredundancy and dimensionality of the fused data vector, $x_{j} \in \mathbb {R}^{N}$, resulting in the compact feature vector, $z_{j} = f(x_{j}) \in \mathbb {R}^{d}$. The matrix $Z=[z_{1} \ldots z_{M}] \in \mathbb {R}^{dxM}$, where $d < N$, can be seen as an embedded or latent representation of the fused data matrix *X*.

### Dataset

In this paper, two datasets are subjected to analysis: the Stavanger University Hospital (SUS) dataset, acquired for the NewbornTime [12] and Neobeat [20] projects, and the Haydom Lutheran Hospital (Haydom) dataset, collected as part of the Safer Birth project^2^. Each dataset consists of annotation text files that correspond to distinct episodes of resuscitation. The SUS dataset encompasses 108 text files, while the Haydom dataset comprises 76 text files. These annotated text files result from manual annotations of video recordings from resuscitation scenarios, utilizing the ELAN video annotation tool. Additionally, the ToB has been manually recorded by an observer writing down the time in second precision at an early stage in the Safer Births project, and by pushing a button in the Liveborn app^3^ in the SUS dataset. The annotation process has focused on the main resuscitation activities, denoted as *C*, performed during these episodes. This partial annotation, carried out by domain experts, serves as training data for ML tasks.

Every annotation text file, $a_{j}$, is structured with four columns of data. The initial column signifies the name of the respective resuscitation activity, $c_i$, while the remaining columns indicate the start, stop, and duration of that particular activity, measured in milliseconds. Our analysis involves extracting information from these annotation text files to visualize the timeline of activities and conduct ML tasks. We focus on the six primary activities within the SUS dataset, denoted as $\{c_1:c_6\}$: *1) baby on table*, *2) drying*, *3) stimulation*, *4) ventilation*, *5) suction*, and *6) chest compression*. Similarly, within the Haydom dataset, we consider seven key activities denoted as $\{c_1:c_7\}$: *1) unwrapped baby*, *2) stimulation*, *3) ventilation*, *4) suction*, *5) attaching dry-electrode ECG (NeoBeat*^4^*)*, *6) wrapped baby*, and *7) chest compression*. For a concise overview of both datasets, refer to Table 2.

**Table 2:** A brief description of the SUS and Haydom datasets

Indicator	SUS Dataset	Haydom Dataset
# of annotation files	108	76
Type of annotation files	Text	Text
Total duration of annotations	14071 sec	53436 sec
Total number$\backslash$ duration of stimulation annotations	532$\backslash$3832 sec	620$\backslash$4193 sec
Total number$\backslash$ duration of ventilation annotations	520$\backslash$5162 sec	342$\backslash$8808 sec
Total number$\backslash$ duration of suction annotations	117$\backslash$1869 sec	197$\backslash$2769 sec
Total number$\backslash$ duration of chest compression annotations	142$\backslash$408 sec	21$\backslash$134 sec

### Proposed periodic encoding methodology

We propose a periodic encoding generator denoted as $G_e(i)$:

4 $$\begin{aligned} G_e(i)=2^i-1 \end{aligned}$$

Let each nonzero element in $g_{i}(n)=G_e(i)$, where *n* denotes the time index, and *i* corresponds to the distinct activities, $c_{i}$.

A distinguishing feature of the proposed generator is its fusion uniqueness property. Specifically, the summation of any two or more generated numbers, even from distinct activities, results in values that do not correspond to any of the individual generated numbers or their fusion:

5 $$\begin{aligned} g_{l}(n)+g_{k}(n) & \ne G_e(i) \;\; \forall \; i,k,l \end{aligned}$$

6 $$\begin{aligned} g_{l}(n)+g_{k}(n) & \ne g_{i}(n)+g_{m}(n) \;\; \forall \; i,k,l,m \end{aligned}$$

To practically generate invariant codes across the chosen activities, our proposed encoding generator follows the subsequent steps:

1. Load each annotation text file for episode $E_{j}$ and read it line-by-line.
2. Group the relevant activities, *C*, from each file and store them in a new container.
3. Arrange the selected activities to maintain an invariant order (example from the SUS dataset): $c_1$=baby on table, $c_2$=drying, $c_3$=stimulation, $c_4$=ventilation, $c_5$=suction, and $c_6$=chest compression.
4. Generate the corresponding feature code vectors, $g_{i}$, for each of the ordered activities, $c_{i}$ based on the duration when the activity is performed. Store the generated feature codes in a matrix $G_{j}$, as described in section Eq. 2.

As an example, Table 3 shows the encoding for 4 activities over a 10-second period of resuscitation, with each number corresponding to one second. Zeros indicate periods where the specific activities where not performed. The methodology for periodic encoding generation can be employed to encode any number $C$ of activities over a given resuscitation time period. Each annotation file is encoded into a matrix $G_j$, which is then stacked into $3^{\text {rd}}$-order tensor $\mathcal {G}$. Refer to Eq. 2 and Fig. 1 for further details.

**Table 3:** Example of encoding of activities over a period of 10 seconds. For each activity, $c_i$, the positive numbers indicate the presence of the activity, while zeros denote its absence. The generated code-vectors $g_2$ and $g_3$ are shown, and last line, x show the fusion of the to activity codes for the episode *j* into vector $x_{j}$

Baby in table $c_1$: $g_{1}$	1	1	1	1	1	1	1	1	1	0
drying $c_2$: $g_{2}$	3	3	3	0	0	0	0	0	0	0
stimulation $c_3$: $g_{3}$	0	0	0	7	7	7	7	0	0	0
ventilation $c_4$: $g_{4}$	0	0	0	0	15	15	15	15	15	0
example $x_{j}=\sum \nolimits _{i} g_{i}$	4	4	4	8	23	23	23	16	16	0

For SUS dataset, the encoding generator, $G_e(n)$, produces 6 unique numbers at each second whenever the activity is being performed. These numbers are {1,3,7,15,31,63} and correspond to the activities listed in the Dataset section, respectively. Similarly, for the Haydom dataset, the encoder generates 7 unique numbers as {1,3,7,15,31,63,127}, corresponding to the activities listed in the Methods section, respectively.

### Resuscitation activity timeline visualization

A timeline provides a visual representation of the resuscitation activities performed on a newborn over time [2]. Resuscitation episodes have different duration. We limit the duration to include the first 12 minutes (720 seconds) of resuscitation, as this includes the most interesting part. The first couple of minutes are the most important for newborn resuscitation. From [9] the duration of the newborn resuscitation episodes plotted in the figures are limited to the first 10 minutes, but it is mentioned that in some examples the resuscitation lasts longer. As we see some activities after 10 minutes in some of our episodes we have limited the duration to 12 minutes, but for most episodes a duration of 8-10 minutes is sufficient. The reason for defining a limit is to have a fixed size vector describing the episode.

The SUS dataset is chosen for visualizing the activities performed on a newborn, primarily because it contains the ToB, which is essential for constructing a reliable timeline. We employed the periodic encoding generator, as discussed in the Proposed periodic encoding methodology section, to produce the corresponding codes for sequentially ordered resuscitation activities, represented by $G$. These activities include ventilation, stimulation, drying, suction, and chest compression. Additionally, we have incorporated an “Baby on table” entry in the timeline visualization. This entry serves to highlight the interval between the ToB and the actual commencement of the resuscitation activities, effectively signaling the onset of the resuscitation procedure.

### Aggregated dataset visualization

We want to illustrate the occurrence of different resuscitation activities denoted as *C*, among all episodes in each dataset. To visualize the occurrence of activities across the aggregated dataset, we employ fusion by addition on the generated codes of the activities, as described in Eq. 3. Specifically, this entails accumulating the codes of resuscitation activities according to the depth, *H*, of the data tensor, $\mathcal {G}$, as depicted in Fig. 1.

The final data matrix, *X*, comprises all fused feature codes, or vectors, over the dataset. $x_{j}\in \mathbb {R}^{N}$ represent the feature code for episode *j*, where the code encapsulate the occurrence of all activities performed at each time point. Subsequently, we use the sparse nearest neighbor graph SNN algorithm [21] to present a visualization of activities across episodes derived from *X*. The SNN is a search method based on constructing a directed graph defined for a set of data points situated in a metric space, such as the Euclidean distance or other types of distances, on a 2-D uniform metric plane.

Utilizing the SNN, visualization is accomplished by mapping sparse points onto a 2-D grid using the indices and values of activities. The computational complexity of the algorithm, specifically for sorting the components of *X*, is $\mathcal {O}(N\log N)$ [22]. In our study, we represent the activities throughout all episodes in the dataset without subjecting the numerical values of *X* to normalization or standardization. By doing so, we bypass data transformations that could potentially displace the centroid of the data samples, adversely affecting the quality of the visualization.

### Dimensionality reduction

Dimensionality reduction can uncover structures and help in understanding the inherent groupings within dataset classes. In this section, we outline the methodology adopted in this paper, showing that learned redundancy reduction is necessary prior to a dimensionality reduction technique for good class separability for our data.

#### Neighborhood component analysis (NCA)

NCA is a dimensionality reduction technique designed to establish a linear mapping from data space, *X*, to an embedding space, *W*, using a learnable Mahalanobis distance metric, proposed to be used with a k-NN clustering scheme [23]. The transformation is done by a learnable matrix *A*.

NCA is employed for dimensionality reduction on both datasets learning a linear mapping for transforming the episodes from the SUS and Haydom datasets into cohesive clusters or groups. By mapping the fused data sample, *X*, as described in Eq. 3, into the mapping space, *W*, NCA optimizes the coordinates of the latent points, $w_j$, within this space. For a given fused data matrix, $X = \{x_j \,|\, x_j \in \mathbb {R}{N},\, j = 1\, ,\dots ,\, M\}$, and class labels vector, *Y*, the NCA algorithm maximizes a metric distance in the mapping space, $W = \{w_j \,|\, w_j \in \mathbb {R}^{d},\, j = 1\, ,\dots ,\, M\}$, between data points belonging to different clusters. That is by finding a learnable linear transformation $\mathbb {R}^N \rightarrow \mathbb {R}^{d}$ ($d < N$) to obtain the mapped space, *W* by $A \in \mathbb {R}^{d \times N}$, $W=AX$.

NCA employs stochastic soft neighbor assignment in the mapping space. This is achieved using a soft probability measure, often referred to as the stochastic selection rule. Consequently, the latent point, $w_j$, in the mapped space, *W*, chooses its neighbor point, $w_k$ using a probabilistic softmax metric, $P_{jk}$, which is grounded on the Euclidean distance:

7 $$\begin{aligned} P_{jk}=\frac{\text {exp}(-||w_j-w_k||^2)}{\sum \nolimits _{l \ne j}{\text {exp}(-||w_l-w_l||^2)} } \end{aligned}$$

The latent point $w_j$ is correctly clustered according to the probability $P_j$:

8 $$\begin{aligned} P_j=\sum \limits _{k \in Y_j} P_{jk} \end{aligned}$$

where $Y_j$ comprises the labels of all input data samples belonging to the same cluster, i.e., $Y_j=\{k|y_j=y_k\}$. As a result, each data sample, $x_j$, represented in a vector is mapped into a latent point, $w_j$, in the mapped space *W*. The conjugate gradient descent optimization [24] maximizes the expected number of latent points (each corresponds to an input vector of activities) assigned to the same class:

9 $$\begin{aligned} \mathcal {L}_{\text {maximize}}=f(A)=\sum \limits _j \sum \limits _{k \in Y_j} P_{jk}=\sum \limits _j P_j \end{aligned}$$

In the experiments described in the Redundancy and dimensionality reduction results section, NCA is employed to reduce the dimensionality and visualize clusters in resuscitation episodes based on encoded annotations. The effectiveness of clustering across the two datasets is demonstrated using labels assigned to the resuscitation episodes, guided by medical professionals and domain experts. First, labels defined by presence or absence of ventilation in an episode is used, representing information that is easy to discover from the encoded episodes. Thereafter labels are based on the newborn’s outcome following the resuscitation procedure, representing information that is not directly encoded. Table 4 outlines the labeling protocol used with the NCA experiments.

**Table 4:** Protocol for labeling episodes for dimensionality reduction and visualization of clusters in the neonatal intensive care Unit (NICU)

Episode Labeling	SUS Dataset	Haydom Dataset
Newborn outcome	Binary labeling (admitted to NICU =1, otherwise=0 )	Multi-class labeling (normal=1, death=2, admitted to NICU=3, stillborn=4)
Ventilation presence	Binary labeling (presence=1, absence=0)	Binary labeling (presence=1, absence=0)

#### Combining learned autoencoder with NCA

AEs are unsupervised models designed to encode and reconstruct data, and they are formed in shallow or deep architectures [25]. AEs share a similar optimization objective of capturing the latent structures of data through reconstruction, benefiting from the characteristics distinct encoding and decoding phases. For a given data sample, $x_i$, in a dataset, $X = \{x_i \,|\, x_i \in \mathbb {R}^N,\, i = 1\, ,\dots ,\, M\}$, the encoding phase produces a mapping $f: \mathbb {R}^N \rightarrow \mathbb {R}^d$, $0< d < N$, to the corresponding encoded data $z_i = f(x_i; \hat{\theta _e})$. The decoding phase produces an inverse mapping $g: \mathbb {R}^d \rightarrow \mathbb {R}^N$, which reconstructs an approximation/ estimation of the input data: $\tilde{x}_i = g(z_{e_{i}}; \hat{\theta _d})$.

Ultimately, the optimization objective is to find the parameters of the encoding and decoding processes that minimize the reconstruction loss:

10 $$\begin{aligned} \min _{\hat{\theta }_e,\, \hat{\theta }_d} \mathcal {L}_{\text {REC}} = \min _{\hat{\theta }_e,\, \hat{\theta }_d} \Vert X- \tilde{X} \Vert _{\text {Err}} \end{aligned}$$

where the reconstruction loss, $\tilde{X}=g(f(X, \hat{\theta _e}),\hat{\theta _d})$, $\text {E}_{\text {rr}}$, is typically measured for the shallow and dense AEs by using the mean square error (MSE) metric. However, in specific applications, other metrics such as Frobenius norm, reconstruction cross-entropy, or $\beta$- divergence can be used [26]. We use MSE as the metric in the loss function in this paper.

**Fig. 2:**
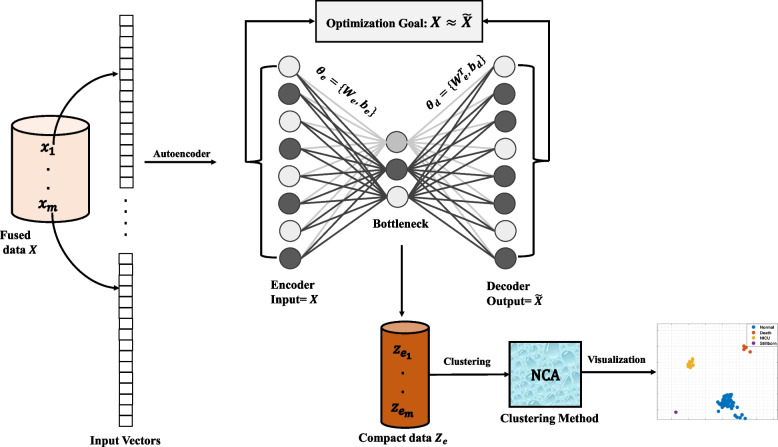
The proposed AE-NCA model. The input data *X* comprises vectors of activities and fused activities, and the AE produces reconstruction $\tilde{X}$ through encoding and decoding parameters, $\theta _{j_{e}}$ and $\theta _{j_{d}}$. The latent space data $Z_e$ of the AE is utilized in the NCA clustering

We propose the AE-NCA model, doing a redundancy reduction through an AE before the final dimensionality reduction and clustering with NCA, introduced in the Neighborhood component analysis (NCA) section. The AE is shallow, composed of three fully connected layers: input, output, and the latent (or bottleneck) layer. The latter captures the latent representations of our resuscitation data vectors, as illustrated in Fig. 2. The size (or dimensionality) of both the input and output layers of the AE matches the size of the input data $x_i$, specifically, $N=720$ neurons, aligning with our fused data vectors. The optimal size of the latent bottleneck layer remains a topic of debate in AE learning. Hence, we adopted the methodologies from [26, 27] to determine the necessary neuron count for the latent space’s dimensionality. Both methods suggest halving the dimensionality of the feature space (input data) and using the resulting value as the dimensionality of the latent space.

In our experiments, the bottleneck layer of the AE used 360 neurons to learn the Haydom data. However, to accommodate the sparsity observed in the SUS data samples that were characterized by a higher proportion of zeros in the data vectors, we increased the dimensionality of the latent space to 500 neurons. This expansion in bottleneck size is necessitated by the data sparsity [26]. All AE experiments were conducted consistently with 4000 epochs, a saturated linear encoder and decoder transfer function, regularization parameter $l_2=0.0001$, sparsity regularizer with coefficient $l=0.01$, learning rate $\eta =0.01$. For consistent experimental reproducibility, we set two distinct random seeds: (seed = 0) for training data with outcome labels, and (seed = 42) for training data based on the presence of ventilation labels.

The loss during learning of the auto-encoder, is calculated as MSE between the input matrix *X* and the resulting $\tilde{X}$. After learning, the final output $\tilde{X}$ is coded back to original values by finding the closest of the allowed values, consisting of the codes 1,3, 7,15,31,63,127 and the sum of combinations of these. As potential misclassifications are done with lower MSEs in the numerical low numbers than in the high numbers, we are considering weighting the loss in future work. However, in our experiments of this paper we can reconstruct correct values after compression for all the episodes, thus there is no actual loss in the compression scheme, only removal of redundant information.

## Results

In this section, we present the results obtained from our experiments. Firstly, we present examples of ToB-based timelines at episode level. Thereafter, our focus shifts to the ensemble of episodes form the two dataset, visualizing different aspects.

### ToB-based timeline visualization

**Fig. 3:**
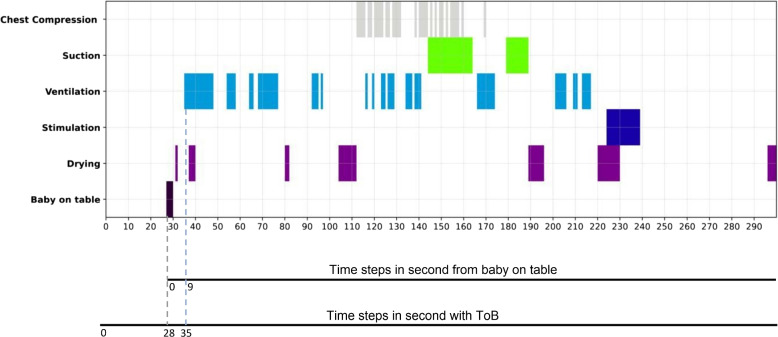
Timeline visualization incorporating the ToB shows the x-axis as time in seconds, while the y-axis enumerates the principal resuscitation activities assessed in our analysis

The activity timeline from the resucsitation table might be the results of manual observations of resuscitations, or based on video from the table, and does not necessarily include ToB observations. To have access to the actual ToB synchronized with the activity timeline at the resuscitation table is crucial to get the proper value of such timeline. Figure 3 depicts an example of a timeline from a resuscitation episode from the SUS dataset, emphasizing the ToB. It also shows the time step (slot) in seconds without considering the ToB.

The ventilation activity, which serves as the main therapeutic resuscitation activity, has been initiated 35 seconds post-birth. This initiation aligns with WHO guidelines, which recommend beginning therapeutic resuscitation activities within the crucial first minute after birth [5].

### Aggregated dataset visualization based on SNN

This section visualizes some results from the aggregated datasets using SNN, as described in the Aggregated dataset visualization section.

**Fig. 4:**
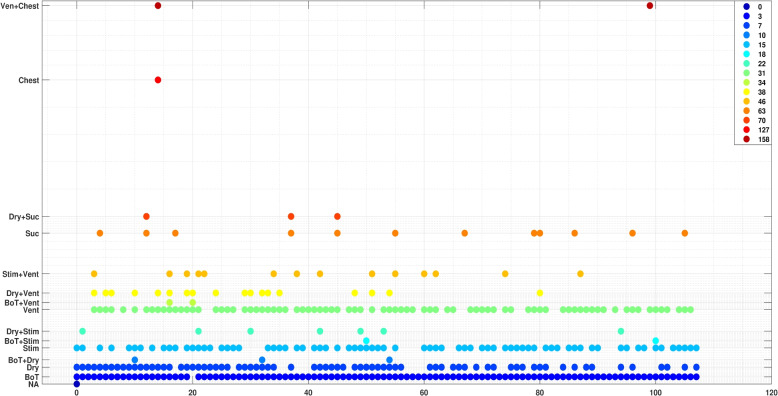
Visualization of episodes from the SUS dataset: the x-axis, represents the index of each annotation text file, while the y-axis features acronyms representing the activities. BOT: Baby On Table, Dry: Drying, Stim: Stimulation, Vent: Ventilation, Suc: Suction, Chest: Chest Compression, and “+” means the existence of these activities performed at the same time. The color bar located in the top-right corner visually translates numerical values from *X* and is associated with the labels on the y-axis

**Fig. 5:**
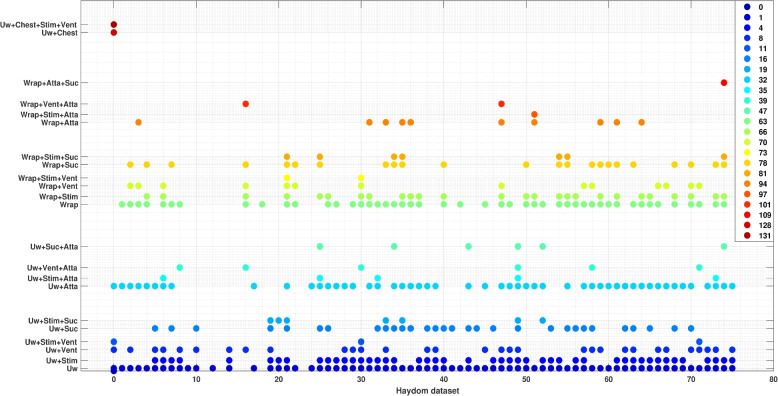
Visualization of episodes from the Haydom dataset: the x-axis represents the index of each annotation text file, while the y-axis features acronyms representing the activities. Uw:unwrapped baby, Stim: stimulation, Vent: ventilation, Suc: suction, Atta:attaching dry-electrode ECG, Wrap:wrapped baby, and Chest:chest compression. “+” means the existence of these activities performed at the same time. The color bar located in the top-right corner visually translates numerical values from *X* and is associated with the labels on the y-axis

Figures 4 and 5 gives a visualization of the existence of resuscitation activities and the fused activities for each episode from the SUS and Haydom datasets, respectively, over the first 12 minutes of resuscitation. In both figures, the horizontal axis (x-axis) represents the index of the annotation text files, corresponding to individual newborn resuscitation episodes. The vertical axis (y-axis) denotes the tags associated with activities and the fused activities observed in each episode.

As can be noticed from both figures, ventilation and stimulation are the predominant resuscitation activities in the SUS dataset, and suction is relatively rare. In contrast, in the Haydom dataset, suction occurs much more frequently, and ventilation is used less frequently, whereas stimulation is used frequently in both datasets. Furthermore, both datasets infrequently implement chest compression. The data was collected under different protocols and cannot be compared directly. Nevertheless it shows some interesting trends and indicates that looking at existence of fused activities might be useful.

### Redundancy and dimensionality reduction results

In this section, we will demonstrate the capability of the AE-NCA method introduced in the Combining learned autoencoder with NCA section to reduce the dimension and visually cluster the fused activities from both datasets consistently. This is compared to using NCA alone.

**Fig. 6:**
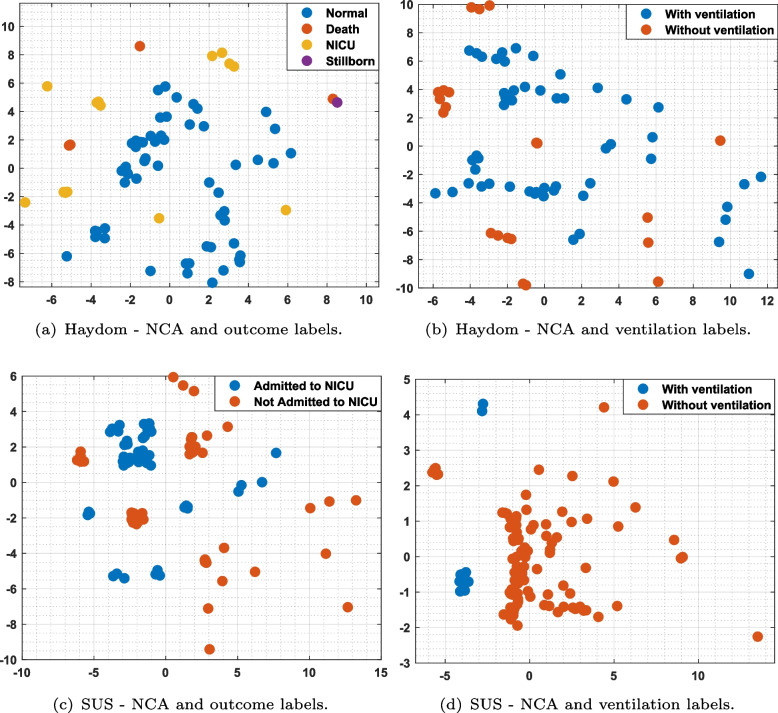
Clustering visualization of the SUS and Haydom datasets. The axes represent the NCA1 and NCA2 features after dimensionality reduction. **a** Haydom dataset employing the outcome labels; **b** Haydom dataset employing the ventilation labels; **c** SUS dataset employing outcome labels; **d** SUS dataset employing the ventilation labels

The data matrix, *X*, is normalized (centered) first and then supplied to the NCA algorithm for clustering based on the labeling protocol reported in Table 4. Figure 6 shows the clustering visualization of the SUS and Haydom datasets from the fused data matrix X. As it can be observed the figures appears cluttered, with no clear indications of clusters or data sample trends. The visualization primarily resembles scatter plots of random points in a 2-D space. The 12 minutes long resuscitation episodes contains redundancies which makes the clustering challenging.

**Fig. 7:**
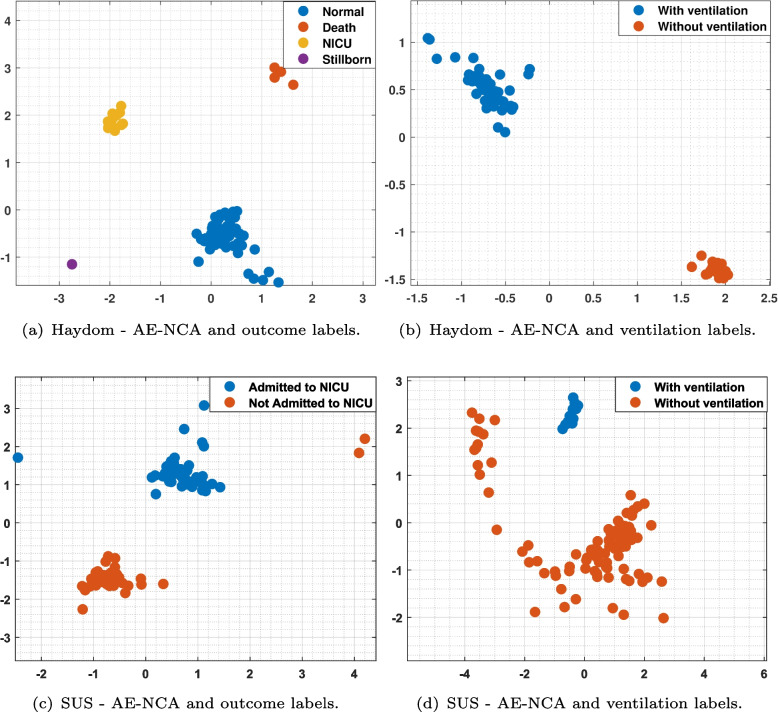
Clustering visualization of our proposed AE-NCA considering the SUS and Haydom datasets.The axes represent the NCA1 and NCA2 features after dimensionality reduction. **a** Haydom dataset employing the outcome labels; **b** Haydom dataset employing the ventilation labels; **c** SUS dataset employing outcome labels; **d** SUS dataset employing the ventilation labels

We introduced the AE-NCA model, which first encodes the data using a fully dense AE providing the latent embeddings, *Z*, and thereafter use NCA for dimensionality reduction and cluster visualization. The results are depicted in Fig. 7. We can observe compact clustering, much more promising than direct clustering of the data matrices *X* in Fig. 6.

The first column in both figure is the most interesting as this is illustrating cluster in the episodes according to the outcome of the resuscitation. If we can learn a dimensionality reduction that gives us clusters with good coherence with the outcome classes, i.e. which infants that dies, has to be admitted to NICU or are fine, that can help identify which factors that are most important for success. The second column in Figs. 6 and 7 is dimensionality reduction learned based on a label we know exist in the fused data matrix *X*,and the episodes could easily have been separated based on a simple test. The purpose of the experiment with these labels is to see how well the visualized clusters performs in terms of as an indicator of the trustworthiness of the more interesting results in column one.

## Discussion

Newborn resuscitation involves a sequence of emergency procedures carried out by pediatricians or midwives to aid newborns who are gasping, not breathing, or exhibiting a weak heartbeat after birth. Moreover, the implementation of these resuscitation procedures adheres to protocols and guidelines established by international entities, such as WHO, and local health authorities. The compliance with the recommended resuscitation protocol can be verified through retrospective analysis of the collected data, which, in our context, encompasses videos, recorded signals, and associated annotation files. We consider our research around newborn resuscitation as exploratory, being a part of one of the largest research networks in the world working on this topic. In the NewbornTime and SaferBirths projects mentioned earlier, data collection is ongoing. We believe that with timelines of many 100s and 1000s of episodes coupled with the status of the newborn before and after, can provide valuable insights on what treatment that works the best, can give debriefing tools, compare practice at different hospitals relative to guidelines etc. Getting such timelines is complicated, collecting objective data, manually annotating or extracting automatically timelines from videos and sensor data. When accurate timelines are available, the next step is to explore and extract interesting knowledge from the data. In this paper we are considering this last step, looking at manually annotated timelines as text files.

The annotation files detail the primary resuscitation activities and their respective duration, as discussed in the Dataset section. The essence of our approach and proposed methods revolves around representing activity occurrences over time via a series of data vectors organized into a multi-dimensional array. As part of this process, we introduced the periodic encoding generator, $G_e(n)$, in the Proposed periodic encoding methodology section. The generator captures periods of activity presence or absence as sequences of distinct codes, with each activity tied to a unique number. This number is repeated for every second the activity is observed and any fusion of numbers, representing simultaneous activities, yields a distinct result, as shown in Table 3. We subjected the resulting data or feature codes, *G*, to various tasks pertinent to newborn resuscitation, including visualization of single episodes and aggregated datasets, redundancy reduction through AE learning, and dimensionality reduction preserving class separability.

In the Resuscitation activity timeline visualization section, we visualized a timeline of resuscitation activities. The Time of Birth is leveraged to display the interval between the exact moment of birth and the commencement of the resuscitation procedure. The timeline offers a visual representation of when resuscitation activities begin and their subsequent duration, and if they are performed in a continuous sequence or with multiple start and stop. Such timelines gives a quantitative description of an episode an can be useful in debriefing tools or in quality improvement tools to study particular episodes, especially if it can be seen in context with the status of the newborn before and after resuscitation. Compliance to guidelines can be found from the timeline description as in Fig. 3, or aggregated with statistics over many episodes.

In the Aggregated dataset visualization section, we presented a visualization method for the entire dataset, summarizing the occurrence of resuscitation activities using the SNN method on generated feature codes. The method accounts for the fusion of activities that occur simultaneously, converting the feature code from a $3^{\text {rd}}$-order tensor $\mathcal {G}$, into a 2-D stacked array, as depicted in Eq. 3. The visual representation adopts a scatter plot format on a 2-D grid. Each point on this grid symbolizes either a specific resuscitation activity or a fusion of concurrent activities, and can be useful to compare different datasets, in quality improvement tools, and to get an overview. The visualizations made in Figs. 4 and 5 can give valuable aggregated insights, like here we can see that there is more suction activity in the Haydom dataset compared to SUS dataset and that chest compression is relatively rare, however note that we want to postpone medical conclusions to a more comprehensive dataset.

The results of the dimensionality reduction experiments in the Redundancy and dimensionality reduction results section show that using NCA directly on the long sequence of activities presented in the fused data-vector $x_{j}$ does not provide visually good clusters. However, when we first use an AE to reduce the redundancy in the vectors, followed by NCA on the feature vectors $z_{j}$, the class separability is much better. The separate clusters in the left column of Fig. 7 indicates that it is possible to find patterns in the activity sequence that strongly correlate with the outcome. i.e. success of the resuscitation. When we have a larger number of episodes, we will further study the episodes to see what the common factors of these groups are, and if we can discover factors that increases the chance of a healthy outcome for newborns with similar status after birth.

### Limitations, challenges, and future work

In the following we list some limitations and challenges and plans for future work:

- *Challenges and Future Investigations on Data Availability:* The datasets derived from both SUS and Haydom, as presented in this study, represent only a fraction of the comprehensive video data amassed during the Safer Births, Neobeat, and NewbornTime studies. The decision to use a subset stems primarily from the labor-intensive nature of manual annotations. Consequently, our current intent is not to derive definitive medical inferences but rather to forge a method that will accelerate the analysis of entire datasets in upcoming ventures. Our primary data source for assessment is the resuscitation table videos, with annotations limited to activities that are straightforwardly distinguishable. This means that certain actions may go unannotated if, for instance, healthcare providers obstruct the view of the newborn or other visual challenges arise. While these annotations are pivotal as ground truth in machine learning applications, they might not provide a full medical representation, where a subset of activities might remain unmarked or have their durations inaccurately noted. In future work, our ongoing efforts in the NewbornTime project are directed toward pioneering automated recognition techniques that can shed light on activity timelines over an expansive set of episodes.
- *Challenges and Future Investigations on the Periodic Generator:* The periodic encoding generator was proposed to produce a succession of data vectors, each mirroring the resuscitation activities. These vectors were specifically based on the durations of individual activities. A notable limitation arises from redundancy within these feature vectors. This redundancy is attributed to the generator’s propensity to assign identical numerical values every second activity is ongoing. To counteract this, the AE-NCA model was formulated, leveraging an AE to reconstruct the original dataset, followed by clustering within the AE’s latent space. However, melding AE with NCA is not without its challenges. The data reconstruction process becomes notably time-consuming. As we move forward, we anticipate alternative methodologies that might either replace or enhance the AE’s role, urging the generator toward the creation of more compact, information-rich codes within the feature vector.
- *Scarcity of previous work:* Analyzing the temporal data of the resuscitation activities performed on a newborn through visualization and clustering is a step towards the optimization of the resuscitation protocol. However, there is hardly any work in the literature to compare with. The collaborative effort through the SaferBirths network, where the NewbornTime project [12] is one of several project, will continue to collect data from different hospitals and propose methods to study these important questions.
- *Lack of clustering metrics and quantitative results:* In future work we will use the method on a more comprehensive dataset. We will do proper clustering following the dimensionality reduction, giving quantitative results in respect to the clustering capabilities for outcome classes and other possible clusters. Classifying with respect to outcome is not a main task but discovering successful resuscitation patterns might come as a consequence.
- *Newborn mortality risk prediction.* The good clustering capability visualized in this paper indicates that a newborn mortality risk prediction score can be partly based on the resuscitation patterns. In future work we wish to look at newborn mortality risk prediction, including knowledge on the health of the mother, information from the labor, like fetal heart rate, labor length, delivery mode, and about the status of the newborn right after birth. Status of the newborn can be measured for example as heart rate or apgar score. With this information as a starting point we can make a risk prediction that updates over time during resuscitation activities.

## Conclusion

This paper is aimed to introduce methods for analyzing newborn resuscitation activity data, through encoding, visualization, aggregation, and dimensionality reduction. The resuscitation data were initially provided as annotation text files, and as a first step an encoding generator with unique codes for combination of activities is proposed. In future work the input can be automatically annotated activity files from video and/or signal data. Dataset aggregation and visualization through SNN can provide insights into, for example, the frequency of activities and variances across different hospitals. Redundancy reduction and dimensionality reduction with the learned AE-NCA method is visually shown to have promising clustering capabilities, indicating a potential for discovering important patterns in resuscitation timelines once the dataset reaches a significant size, and for developing risk prediction scores in future work.

## Data Availability

The datasets are private and not available due to ethical approval restrictions.

## Abbreviations

AE: Autoencoder
AI: Artificial intelligence
CAP: Positive airway pressure
KNN: K-nearest neighbour
LLO: Leave one out
ML: Machine learning
NCA: Neighborhood component analysis
NICU: Neonatal intensive care Uni
PPV: Positive Pressure Ventilation
SNN: sparse nearest neighbor graph
WHO: world health organization
ToB: Time of Birth
SUS: Stavanger University Hospital

## Footnotes

1 https://www.uis.no/newborntime

2 https://saferbirths.com/

3 Liveborn app is developed by Laerdal medical AS

4 https://shop.laerdalglobalhealth.com/product/neobeat/

## Code availability

Upon request.
